# Low Numbers of Vascular Vessels Correlate to Progression in Hormone-Naïve Prostate Carcinomas Undergoing Radical Prostatectomy

**DOI:** 10.3390/cancers11091356

**Published:** 2019-09-12

**Authors:** Julia Smentoch, Jolanta Szade, Anna J. Żaczek, Elke Eltze, Axel Semjonow, Burkhard Brandt, Natalia Bednarz-Knoll

**Affiliations:** 1 Laboratory of Cell Biology, Department of Medical Biotechnology, Medical University of Gdańsk, Gdańsk 80-211, Poland; julia.smentoch@gumed.edu.pl (J.S.);; 2 Department of Pathomorphology, Medical University of Gdańsk, Gdańsk 80-214, Poland; jszade@gumed.edu.pl; 3 Institute of Pathology Saarbruecken-Rastpfuhl, Saarbruecken 66113, Germany; e.eltze@pathologie-saarbruecken.de; 4 Department of Urology, Prostate Center, University Clinic Münster, Münster 48149, Germany; axel.semjonow@ukmuenster.de; 5 Institute of Clinical Chemistry, University Medical Centre Schleswig-Holstein, Kiel 24105, Germany; burkhard.brandt@uksh.de

**Keywords:** prostate cancer, vascular vessels, lymphatic vessels, CD34, podoplanin, hormone-naïve patients, tumor progression, heterogeneity

## Abstract

Vascularization influences tumor development by supporting the nutrition and dissemination of tumor cells. On the other hand, a low number of vascular vessels (VV^low^) may induce hypoxia, accounting for selection of resistant clone(s) of tumor cells. This study aimed to evaluate the prognostic significance of vascular (VV) and lymphatic vessels (LV) in prostate cancer (PCa). Tumor samples from 400 PCa patients undergoing radical prostatectomy (RP) were prepared in duplex as tissue microarrays. Numbers of VV and LV were evaluated using immunohistochemistry detecting CD34 and podoplanin, respectively, and correlated to clinical data, biochemical recurrence (BR), and proteins analyzed in tumor cells. VV^low^ and LV were found in 32% and 43% of patients with informative PCa samples, respectively. VV^low^ correlated with a shorter time to BR 3, 5, and 10 years after RP in hormone-naïve patients (*p* = 0.028, *p* = 0.027 and *p* = 0.056, respectively). It was also shown to be an independent prognostic factor 5 years after surgery (multivariate analysis, *p* = 0.046). Tumors characterized by VV^low^ expressed the epithelial cell adhesion molecule, EpCAM, less frequently (*p* = 0.016) and revealed a borderline correlation to increased levels of tumor cell invasion marker Loxl-2 (*p* = 0.059). No correlations were found for LV. In summary, VV^low^ in hormone-naïve patients undergoing RP has prognostic potential and seems to be related to an aggressive phenotype of tumor cells.

## 1. Introduction

Prostate cancer (PCa) is one of the most commonly diagnosed cancer types among men in the industrialized countries [1]. Early diagnosis has increased since the introduction of serum prostate-specific antigen (PSA) screening. Nevertheless, determination of the risk of recurrence is still incomplete and further research is required to better understand the metastatic PCa cascade. Biochemical recurrence (BR) is still assumed to be the earliest indicator of patient relapse. It has been estimated that approximately 35% of patients manifest BR within 10 years after surgery and overt metastases approximately 8 years after BR [2]. Further research is needed to identify markers relevant for individualized prognostication and tailoring of therapy.

(Neo)vascularization is considered as one of the putative factors influencing tumor development and in consequence impacting patients’ outcome in different solid tumors including PCa [3,4,5,6,7,8]. Rich vascularity might guarantee appropriate nutrition of tumor cells and putatively facilitate their dissemination. On the other hand, a low number of vascular vessels may result in poor oxygenation, i.e., hypoxia. In PCa, hypoxia occurs even at early stages of disease [9] and is associated with poor prognosis due to the selection of the most resistant clone(s) of tumor cells [9]. It was shown to induce and/or maintain metastases-promoting processes such as epithelial-to-mesenchymal transition (EMT), stemness, cell survival, and proliferation [9,10].

To date, the relationship between vascular (VV) and/or lymphatic vessels (LV), and patient outcome in PCa has not been fully clarified. Previous studies showed variable outcomes (i.e., correlations to clinico-pathological parameters and patient survival or lack of) depending on the study design and applied methodology (including origin and type of PCa specimens, type of targeted proteins to detect endothelial cells, area of analysis, type of treatment, etc.) [11]. Erbesdobler et al. performed a study on the highest (to the best of our knowledge) number of prostatectomy specimens (*n* = 3261), showing the correlation between higher VV and disease aggressiveness as well as progression [12]. As far as we know, data on low vascularity potentially inducing hypoxia are not present in the literature (or do not reach statistical significance) and different therapeutic regimen are scarcely described [13], and androgen deprivation therapy (ADT) might potentially impact angiogenesis [14].

Therefore, in the current study the association between vasculature and the aggressive PCa phenotype and disease progression was investigated in unselected PCa patients, hormone-naïve patients, and those treated with neoadjuvant ADT. CD34 and podoplanin, two proteins commonly used for detection of VV and LV, respectively, were assessed in order to examine the number of vessels, as well as to examine their heterogeneity and elucidate putative clinical relevance. Of note, in contrast to the majority of studies, we also considered minimal vessel numbers and their possible role in tumor progression or the relationship to clinical parameters.

## 2. Results

### 2.1. High Variability of Numbers of Vascular and Lymphatic Vessels in Prostate Carcinoma

**Figure 1:**
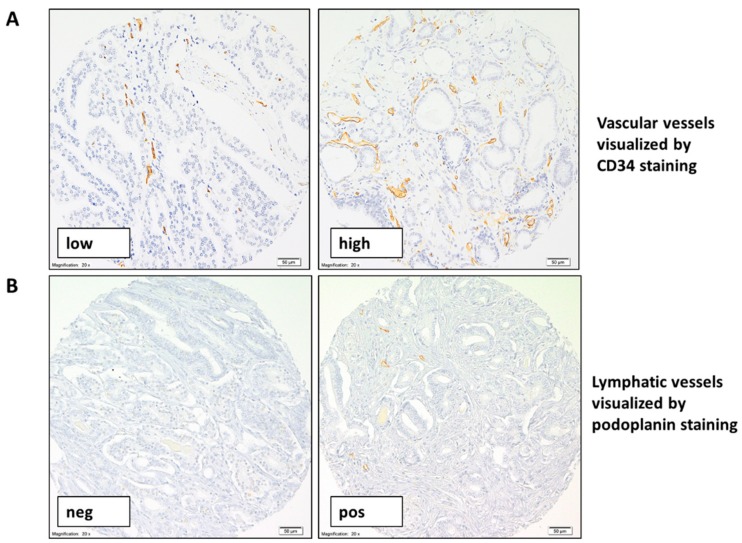
Representative pictures of CD34 and podoplanin staining in prostate cancer (PCa). Representative pictures of CD34 (**A**) and podoplanin (**B**) immunohistochemical staining (brown) in PCa with different number of identified vascular and lymphatic vessels, respectively (magnification 200×).

In total, 699 tumor samples from 382 patients and 709 tumor samples from 388 patients were informative for CD34 and podoplanin staining (Figure 1A,B), respectively. All examined tumor samples had a diameter of 0.6 mm (i.e., an area of 0.28 mm^2^) and contained between 10 and 1000 tumor cells [15]. Of note, none of the detected VV and LV were invaded by tumor cells.

CD43-positive VV (Figure 1A) were identified in almost all examined tumor samples with the mean total number of VV determined as 11 and a range of 1 to 62 VV per tumor fragment. Only five tumor samples were characterized by a complete lack of VV.

minVV and maxVV (i.e. minimal or maximal VV, Figure S1) were tested to categorize the patients for further statistical analysis (Figure S1). However, only minVV dichotomized according to the lower quartile (equal 3 VV) into minVV^low^ and minVV^high^ correlated to clinical outcome, and therefore this classification is described in the current study in detail. According to this classification system, 32% (*n* = 123) patients were characterized by minVV^low^.

Podoplanin-positive LV (Figure 1B) were detected in 194 (27%) of 709 tumor samples with the mean and median total number of LV determined as 1 and ranging from 1 to 16 LV per tumor fragment. Overall, 515 tumor samples did not have any LV.

minLV and maxLV were also tested to categorize the patients for further statistical analysis (Figure S1). However, as there were no correlations to clinical data for LV, any positive maxLV was categorized as LV^pos^, whereas no LV was termed LV^neg^. In total, 167 (43%) patients were defined as LV^pos^ according to this classification system.

Heterogeneity in number of vessels (i.e., the opposed numbers of vessels, low vs. high for VV or negative vs. positive for LV, in two examined and informative tumor samples) was found in approximately one-third of all patients informative for investigated type of vessels in two tumor samples (Supplementary Table S1). There was no difference in the distribution of number of VV and LV or their variability between mono- and multifocal PCa (Supplementary Tables S1 and S2).

### 2.2. Clinical Relevance of Low Number of Vascular Vessels

Different cut-offs (i.e., mean, median, quartiles) to categorize the outcomes and clinical subgroups of the study cohort (i.e., unselected cohort, hormone-naïve patients, and patients treated with neoadjuvant ADT) were tested in order to determine putative relevance of vascular and lymphatic vascularity in PCa. No correlations were found between vasculature and clinico-pathological parameters (presented outcomes for minVV and maxLV are described in Supplementary Table S2).

**Figure 2:**
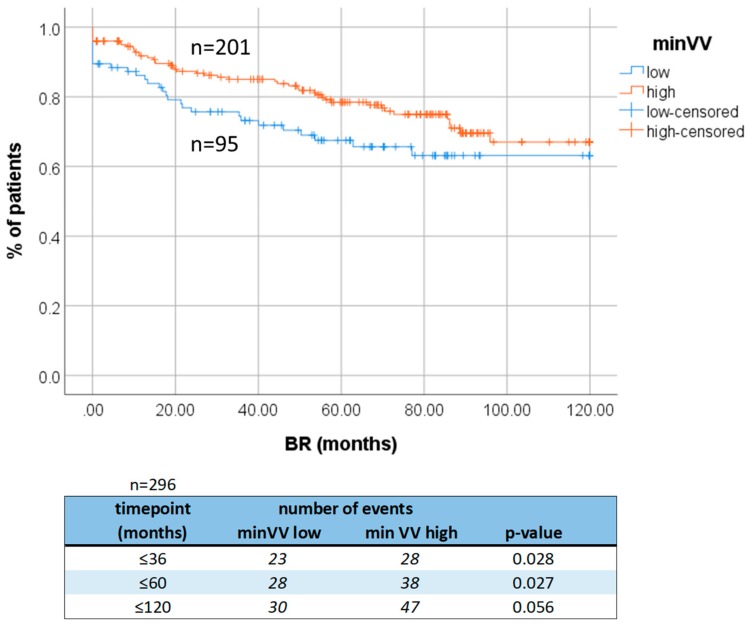
Survival analysis. Association of minVV to shorter time to biochemical recurrence in hormone-naïve PCa patients. BR: biochemical recurrence. VV indicates vascular vessels.

minVV^low^ correlated with the shorter time to BR (Kaplan-Meier plot, *n* = 296) at timepoints of 3, 5, and 10 years after surgery (*p* = 0.028, *p* = 0.027, and *p* = 0.056, respectively; Figure 2) in the hormone-naïve patients. It appeared to be an independent prognostic factor in this cohort of patients in the multivariate analysis including T status, Gleason score, and minVV status 5 years after surgery (*n* = 296, *p* = 0.046, HR—hazard ratio 0.607, 95% CI 0.372–0.991, Table 1).

### 2.3. Signatures of Aggressive Molecular Phenotype in Tumors Characterized by Low Number of Vascular Vessels

The panel of different PCa aggressiveness- and progression-related proteins was assessed before in tumor cells of individual tumor samples of the examined patients: Ki-67, apoptosis marker (ApopTag), cytokeratins (CK5/6, CK14, CK8/18, CK19), vimentin, E- and N-cadherin, aldehyde dehydrogenase 1 (ALDH1), epidermal growth factor receptor (EGFR), epithelial cell adhesion molecule (EpCAM), B-cell lymphoma-2 apoptosis regulator (Bcl-2), and lysyl oxidase homolog 2 (Loxl-2) [16,17]. The numbers of VV were compared to those proteins in the individual tumor samples (Supplementary Table S3).

**Figure 3:**
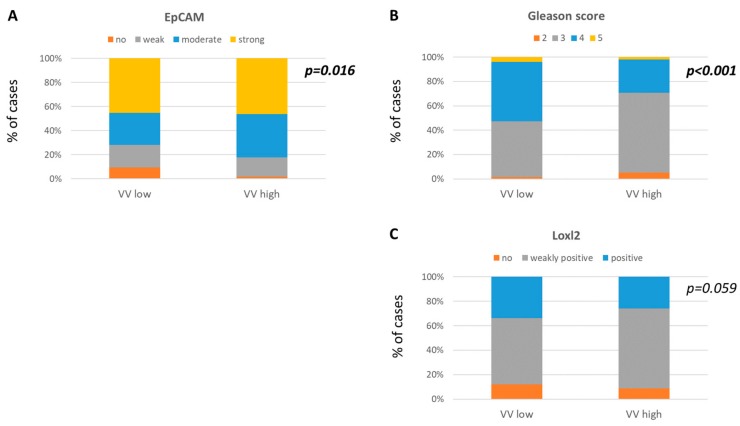
Molecular characteristics of PCa in comparison to VV status. VV^low^ correlations to less frequent (**A**) EpCAM expression, (**B**) higher Gleason score, and borderline correlation to more frequent (**C**) Loxl-2 expression.

Tumors with VV^low^ not subjected to any preoperative ADT (i.e., tumors of hormone-naïve subcohort of patients) less frequently expressed the epithelial cell marker EpCAM in tumor cells (*n* = 296, Chi-squared = 10.314, *p* = 0.016, Figure 3A) and were usually characterized by the higher Gleason score (*n* = 585, Chi^2^ = 25.116, *p* < 0.001, Figure 3B). In addition, VV^low^ revealed a borderline correlation to the increased presence of a hypoxia-related marker of tumor invasion on the extracellular matrix, Loxl-2, in tumor cells (*n* = 558, Chi-squared = 5.669, *p* = 0.059, Figure 3C).

Interestingly, when only the lower and upper quartiles of VV were compared (i.e., groups of tumors with VV < 5 and VV > 15, respectively), the tumors with low vasculature were characterized significantly more frequently by Loxl-2 (*p* = 0.013), proliferation marker Ki-67 (*p* = 0.034), and the apoptosis marker (*p* = 0.022; data not shown [18]).

## 3. Discussion

Tumor (neo)vasculature might play a crucial role in tumor development and progression. Here, we show for the first time that low vasculature correlates to worse clinical outcome in hormone-naïve PCa patients after radical prostatectomy and is associated with aggressive phenotype of tumor cells.

The number of vascular and lymphatic vessels is considered as a significant microenvironmental factor impacting tumor fate in different solid tumors [4,5,7,19]. However, inconsistent study designs and applied methodologies (i.e., different origin, type and area of the analyzed PCa specimens, type of targeted proteins to detect endothelial cells, etc.) result in different outcomes showing both correlations and their lack between number of vessels and clinico-pathological parameters in PCa [11]. In the current study, two commonly used proteins, CD34 and podoplanin, were examined to define the numbers of VV and LV, respectively. Our evaluation method was similar to commonly used so called Weidner method [20]. However, the applied study approach allowed for an examination of the clinical significance of both minimal and maximal numbers of vessels detected within the defined fragments of tumors (area equal 0.28 mm^2^) without an a priori assumption that only high vasculature might support tumor progression. Of note, tissue microarrays (TMAs) used in this study represented a collection of potentially different tumor samples of individual patients prepared in order to study PCa heterogeneity. In addition, to exclude bias caused potentially by ADT [14], clinical relevance of vasculature was compared in the unselected cohort, as well as hormone-naïve PCa patients and those treated with ADT preoperatively.

VVs were detected in almost all samples, whereas LVs were found in only 27% of tumor samples. Intratumoral heterogeneity of VV and LV numbers was observed in approximately one-third of the patients, which substantiates the high heterogeneity described in PCa for many factors. VV^low^ and LV were found in 32% and 43% of patients, respectively. Only minVV^low^ indicating the lowest numbers of vascular vessels (i.e., the lower quartile of minimal values assigned for a patient) correlated to worse clinical outcome in hormone-naïve PCa patients in the timeframe of 3–10 years after surgery. Of note, in the current study minVV^low^ was an independent prognostic factor in the multivariate analysis, substantiating its prognostic potential in patients without ADT. We did not observe any correlation to clinical outcome in the cohort of unselected PCa patients or for maxVV and LV, nor for minVV in the PCa patients undergoing ADT before the prostatectomy. However, this cohort was relatively small (*n* = 67) which might have biased the outcome. Those results seem to be counterintuitive as the other groups showed that the higher number of VV [12,21,22,23] and also the presence of LV may increase the risk of BR in the patients undergoing radical prostatectomy after neoadjuvant treatment with androgen blockage [24], correlating with high Gleason score and other clinico-pathological parameters indicating advanced disease [12,21,22,23,25,26,27]. In particular, Erbersdobler et al. showed the clinical significance of high number of vessels in, to the best of our knowledge, the largest study on 3261 prostatectomy specimens prepared as TMA [12]. However, they did not confirm this observation in the multivariate analysis [12] and Mucci et al. did not observe a correlation between high number of VV and cancer-specific mortality [26]. Of note, a high number of VV evaluated using CD105, another protein detecting endothelial cells, was also identified as a significant and independent predictor of biochemical recurrence in prostate cancer patients who underwent radical prostatectomy with ADT [13]. Our results and those of the literature could suggest that vascularization develops differentially under different androgen conditions, regulating tumor development and patient outcome in a different way. In addition, the majority of the studies focused on the so-called “hot spot” with the highest number of VV. This approach might be also biased [28] and result in exclusion of fragments of tumor with low number of vessels, potentially still crucial for progression but driven by different biological mechanisms.

Indeed, in our study tumors characterized by VV^low^ exerted some features of more a aggressive phenotype of tumor cells. They were characterized more frequently by the absence of the epithelial cell marker EpCAM [29], which might suggest that those tumors undergo EMT known to facilitate migration, and even induce stemness [30]. They also more frequently expressed higher levels of Loxl-2, a protein related to invasion of tumor cells on extracellular matrix, and in comparison to highly-vascularized tumors this was also the case for proliferation marker Ki-67 and the apoptosis marker. All those proteins are known to correlate to poorer clinical outcome both in PCa and/or other tumor entities [31,32,33]. Of note, they might be expressed under hypoxic conditions expected to occur in less vascularized samples of tumors [32]. Hypoxia-related markers (such as hypoxia-inducible factor 1-alpha, HIF-1α) were not investigated in this study. However, it might be still speculated that hypoxia occurred in the examined tumors with low numbers of VV, potentially promoting a proliferation/apoptosis imbalance as well as induction of EMT [34] and acquisition of more aggressive phenotype by tumor cells [30]. In concordance, Loxl-2 was shown to be upregulated by HIF-1α in a hypoxic tumor microenvironment in hepatocellular cancer [35], and to induce EMT in colorectal and breast carcinomas [36,37].

## 4. Material and Methods

### 4.1. Patient Cohort

Tumor samples from 400 PCa patients were collected following radical retropubic prostatectomy at the Department of Urology at the University Clinic Münster (Germany) after patients gave informed consent, and were prepared in duplex as 2 TMAs as described previously [15,16]. Briefly, two tumor samples were selected for each patient from different areas of the tumor (if the tumor was monofocal) or different tumor foci (if the tumor was multifocal). Patients were characterized by different clinico-pathological parameters (Table 2) and different molecular phenotypes of tumor cells (Supplementary Table S4). Measurement of serum PSA concentration was performed by two methods: (1) up to 2009 using Tandem-E (Hybritech, San Diego, CA, USA) and (2) from 2010 using Access 2 (Hybritech-calibrated, Beckman Coulter, Brea, CA, USA). BR was defined as two consecutive concentrations of PSA above 0.1 ng/mL. The time point of BR was defined as the first PSA concentration above 0.1 ng/mL. The last follow-up was performed in June 2019 (mean time to BR was 60 months, range 0–201 months).

Sixty-nine of the patients received neoadjuvant ADT (i.e., before surgery), the remaining 331 patients had no hormonal manipulations prior to BR and were termed hormone-naïve. Those two subcohorts were considered separately for statistical analysis. The study was approved by the local Ethics Committee (Ethik Kommission der Aerztekammer Westfalen-Lippe und der Medizinischen Fakultaet der Westfaelischen Wilhelms-Universitaet Muenster, Germany, nr 2007–467–f–S).

### 4.2. Immunohistochemistry for CD34 and Podoplanin

Immunohistochemical staining was performed on TMA sections (4–5 µm thick) using commercially available ready-to-use mouse monoclonal anti-CD34 antibody (clone QBEnd10, Agilent Dako, Santa Clara, CA, USA) for VV and anti-podoplanin antibody (clone REF 760–4395, Roche, Switzerland) for LV visualized by EnVision FLEX+ system (Dako) and UltraView DAB Benchmark XT (Roche) system, respectively.

### 4.3. The Evaluation of VV and LV Density

The immunohistochemical staining was evaluated at the 200× magnification using light microscope (Olympus BX 43, Olympus, Japan). The total number of CD34- or podoplanin-positive VV or LV, respectively, with visible light of lumen was documented in each informative tumor sample (0.6 mm diameter, area 0.28 mm^2^).

In order to examine the possible variability of numbers of vessels, vessels were counted separately in two tumor samples of each patient. The tumor sample with the lower number of vessels was assigned minVV or minLV, the sample with the higher number of vessels was considered as maxVV or maxLV (Figure S1). If only one tumor sample was informative, the vessel count of that tumor sample was assigned to the patient. The assigned discrete values for minVV, maxVV, minLV, and maxLV were dichotomized based on different mathematical cut-offs (i.e., mean, median, quartiles) and compared to clinical data and patient survival (Figure S1).

### 4.4. Immunohistochemistry for Tumor Cell Markers

Immunohistochemical staining for Ki-67, apoptosis marker (ApopTag), cytokeratins (CK5/6, CK14, CK8/18, CK19), vimentin, E- and N-cadherin, ALDH1, EGFR, EpCAM, and Loxl-2 protein was performed, evaluated, and categorized as negative or positive (and for Loxl-2 as negative, weakly positive, or positive) as described ([16,17,38,39], brief description in Supplementary Table S5).

Immunohistochemical staining for Bcl-2 (dilution 1:250; clone124, DAKO, Denmark) was performed using Autostainer (Dako), and alkaline phosphatase detection kit (Universal LSAB™ Kit/HRP, Rabbit/Mouse/Goat, Dako, Denmark). Staining was categorized based on the intensity as negative (i.e., no or weak expression), or positive (i.e., moderate to strong expression).

### 4.5. Statistical Analysis

Statistical analysis was performed using SPSS software (IBM) version 25 licensed to the University of Gdańsk. The comparison of number of vessels and clinical data or proteins detected in tumor cells was performed using Chi-squared or Fisher’s exact tests. The association between number of vessels and time to BR was calculated using Mantel Cox test and presented using Kaplan–Meier plots. The uni- and multivariate analysis was performed using the Cox-Hazard-Potential regression model (95% CI). The obtained results were considered statistically significant at *p* < 0.05. Cases with missing data were excluded from the statistical analysis.

## 5. Conclusions

In summary, tumors with VV^low^ seem to have a more aggressive phenotype and shorter time to BR in hormone-naïve PCa, suggesting the pivotal role of vascular vessels in regulation of tumor progression. However, further studies are needed to dissect this process in detail.

## Figures and Tables

**Table 1:** Multivariate analysis. Statistically significant results are bolded. PSA: prostate-specific antigen, minVV^low^—low number of vascular vessels, minVV^high^—high number of vascular vessels.

5 Years	Univariate Analysis			Multivariate Analysis		
	*p*-Value	HR	95% CI	*p*-Value	HR	95% Cl
**T3–4 vs. T1–2**	<0.001	3.734	2.068–6.742	<0.001	3.337	1.815–6.136
**N1–2 vs. N0**	0.260	1.691	0.678–4.251	-	-	-
**Preoperative PSA ≥ 4 ng/mL vs. < 4 ng/mL**	0.076	5.971	0.829–43.030	-	-	-
**Age (≥ 64 vs. < 64)**	0.515	1.174	0.724–1.903	-	-	-
**Gleason scale ≥ 7 vs. < 7**	0.010	2.796	1.277–6.120	0.190	1.716	0.765–3.849
**minVV^low^ vs. minVV^high^**	0.031	0.584	0.358–0.951	0.046	0.607	0.372–0.991

**Table 2:** Distribution of clinical parameters in the study cohort. neg: negative, pos: positive. Note that not all numbers sum up to 400 due to the missing data. ADT: androgen deprivation therapy.

Clinical and Pathological Parameters	Status	*n*	%
Age (years)	<median (64)	208	52.00
	≥median (64)	192	48.00
	total	400	
T status	T2	3	0.80
	T3a	184	46.10
	T3b	190	47.60
	T4	22	5.50
	total	399	
N status	N0	367	94.60
	N1	21	5.40
	total	388	
Gleason score sum	<7	102	25.50
	7	253	63.20
	>7	45	11.30
	total	400	
Preoperative PSA	<4 ng/mL	42	10.80
	4–10 ng/mL	171	44.00
	10–20 ng/mL	118	30.30
	>20 ng/mL	58	14.90
	total	389	
d’Amico scale	low risk	15	3.90
	intermediate low risk	91	23.50
	intermediate high risk	246	63.60
	high risk	35	9.00
	total	387	
Preoperative ADT	neg	331	82.80
	pos	69	17.30
	total	400	
Biochemical recurrence	no	293	73.60
	yes	105	26.40
	total	398	
Tumor focality	monofocal	69	17.30
	multifocal	329	82.70
	total	398	

## Supplementary Materials

The following are available online at https://www.mdpi.com/2072-6694/11/9/1356/s1, Figure S1: Scheme showing the process of evaluation of tumor samples and categorization of the outcomes, Table S1: Heterogeneity of VV and LV numbers in PCa, Table S2: Comparison of VV (A) and LV (B) numbers to clinico-pathological parameters in the whole cohort, hormone-naïve patients, and patients treated with preoperative ADT, Table S3: Associations of minVVlow to selected proteins in tumors without preoperative androgen deprivation therapy, Table S4: Molecular marker distribution. Distribution of the selected molecular signatures in tumor samples from unselected PCa patients cohort, Table S5: Details of immunohistochemistry for proteins analysed in tumor cells.
