# Supplementary material for: Low Numbers of Vascular Vessels Correlate to Progression in Hormone-Naïve Prostate Carcinomas Undergoing Radical Prostatectomy
Source: Cancers (Basel). 2019 Sep 12;11(9):1356. doi: 10.3390/cancers11091356 (PMC6770894; doi:10.3390/cancers11091356)
Supplement: Supplementary file 1 [file cancers-11-01356-s001.pdf]

Supplementary Materials

Table S1. Heterogeneity of VV and LV numbers in PCa. VV and LV numbers heterogeneity among mono- and multi-focal PCa. Note that not all numbers sum up to 400 due to the missing data. Mono indicates monofocal PCa, multi—multifocal PCa, pts—patients.

Vascular vessels							
**Tumor Focality**	**No. of informative tumor samples/patient**	**Total no. of patients**	**Number of patients**				
			**minVV low**	**minVV high**	**minVV low/ minVV high**	**minVV low/ minVV low**	**minVV high/ minVV high**
Mono	1	5	0	5			
	2	58			19	0	39
					homogeneity, total n = 39 pts (67%)		
					heterogeneity, total n = 19 pts (33%)		
Multi	1	60	17	43			
	2	259			75	12	172
					homogeneity, total n = 184 pts (71%)		
					heterogeneity, total n = 75 pts (29%)		
Total	patients	382					
	tissue cores	699					
**Lymphatic vessels ***							
**Tumor Focality**	**No. of informative tumor samples/patient**	**Total no. of patients**	**Number of patients**				
			**LV neg**	**LV pos**	**LV neg/ LV neg**	**LV neg/ LV pos**	**LV pos/ LV pos**
Mono	1	6	6	0			
	2	57			32	18	7
					homogeneity, total n = 39 pts (68%)		
					heterogeneity, total n = 18 pts (32%)		
Multi	1	60	37	23			
	2	263			145	98	20
					homogeneity, total n = 165 pts (63%)		
					heterogeneity, total n = 98 pts (37%)		
Total	patients	386					
	tissue cores	706					

* In case of podoplanin 2 additional patients did not have the information about the focality but was includede in statistical analysis in comparision to other clinico-pathological parameters

Table S2. Comparison of VV (A) and LV (B) numbers to clinico-pathological parameters in unselected cohort of PCa patients, hormone-naïve patients and patients treated with preoperative ADT.

**A) VV**													
**Clinical and pathologic parameters**	**Status**	**Whole cohort, minVV**				**Hormone-naïve patients, minVV**				**Androgen deprivation therapy, minVV**			
		**low**		**high**		**low**		**high**		**low**		**high**	
		**n**	**%**	**n**	**%**	**n**	**%**	**n**	**%**	**n**	**%**	**n**	**%**
Age (years)	< median (64)	61	49.60	140	53.20	51	49.00	113	52.80	10	52.60	27	55.10
	> = median (64)	62	50.40	123	46.80	53	51.00	101	47.20	9	47.40	22	44.90
	p value	*p* = 0.505				*p* = 0.528				*p* = 1.000 (F)			
T status	T2	0	0.00	3	1.10	0	0.00	2	0.90	0	0.00	1	2.10
	T3a	53	43.10	126	48.10	47	45.20	108	50.50	6	31.60	18	37.50
	T3b	63	51.20	118	45.00	51	49.00	95	44.40	12	63.20	23	47.90
	T4	7	5.70	15	5.70	6	5.80	9	4.20	1	5.30	6	12.50
	p value	*p* = 0.468				*p* = 0.559				*p* = 0.611			
N status	N0	113	95.00	241	94.50	96	95.00	193	93.70	17	94.40	48	98.00
	N1	6	5.00	14	5.50	5	5.00	13	6.30	1	5.60	1	2.00
	p value	*p* = 1.000 (F)				*p* = 0.798 (F)				*p* = 0.468 (F)			
Gleason score	< 7	28	22.80	70	26.60	26	25.00	59	27.60	2	10.50	11	22.40
	7	79	64.20	166	63.10	64	61.50	136	63.60	15	78.90	30	61.20
	> 7	16	13.00	27	10.30	14	13.50	19	8.90	2	10.50	8	16.30
	p value	*p* = 0.585				*p* = 0.442				*p* = 0.371			
Preoperative PSA	< 4 ng/ml	16	13.40	24	9.40	16	15.70	20	9.50	0	0.00	4	8.90
	4–10 ng/ml	56	47.10	108	42.20	49	48.00	85	40.30	7	41.20	23	51.10
	10–20 ng/ml	31	26.10	82	32.00	26	25.50	71	33.60	5	29.40	11	24.40
	> 20 ng/ml	16	13.40	42	16.40	11	10.80	35	16.60	5	29.40	7	15.60
	p value	*p* = 0.372				*p* = 0.097				*p* = 0.376			
d'Amico scale	low risk	3	2.50	12	4.70	3	3.00	10	4.80	0	0.00	2	4.30
	intermediate low risk	29	24.20	59	23.20	25	24.80	51	24.50	4	21.10	8	17.40
	intermediate high risk	77	64.20	159	62.60	64	63.40	131	63.00	13	68.40	28	60.90
	high risk	11	9.20	24	9.40	9	8.90	16	7.70	2	10.50	8	17.40
	p value	*p* = 0.783				*p* = 0.879				*p* = 0.692			
Preoperative ADT	no ADT	104	84.60	214	81.40	-	-	-	-	-	-	-	-
	ADT	19	15.40	49	18.60	-	-	-	-	-	-	-	-
	p value	*p* = 0.444				-				-			
	yes	36	29.30	66	25.20	30	28.80	50	23.50	6	31.60	16	32.70

Biochemical recurrence	no	87	70.70	196	74.80	74	71.20	163	76.50	13	68.40	33	67.30
	p value	*p* = 0.398				*p* = 0.301				*p* = 1.000 (F)			
Tumor Focality	mono	19	15.40	44	16.90	18	17.30	34	16.00	1	5.30	10	20.40
	multi	104	84.60	217	83.10	86	82.70	178	84.00	18	94.70	39	79.60
	p value	*p* = 0.728				*p* = 0.775				*p* = 0.163 (F)			

Table S3. Associations of minVV^{low} to selected proteins in tumors without preoperative androgen deprivation therapy. Comparison of VV status to selected proteins in individual fragments of tumors of hormone-naïve PCa patients. The statistical tests were performed using Chi square test or Fisher exact test (F). minVV^{low} indicates low minimal number of vascular vessels, minVV^{high} - high minimal number of vascular vessels, E-cad – E-cadherin, N-cad – N-cadherin, Vim – vimentin, neg – negative, pos – positive.

**Molecular marker**		**CD34-positive vessels**			
		**VV^{low}**		**VV^{high}**	
		**n**	**%**	**n**	**%**
**CK5/6**	neg	113	86.90	332	86.90
	pos	17	13.10	50	13.10
	Total	p = 0.997			
CK14	neg	94	71.20	261	70.00
	pos	38	28.80	112	30.00
	Total	P = 0.789			
CK8/18	neg	46	33.30	144	35.70
	pos	92	66.70	259	64.30
	Total	P = 0.610			
CK19	neg	67	51.90	205	53.70
	pos	62	48.10	177	46.30
	Total	P = 0.734			
EpCAM	neg	21	28.00	39	17.60
	Pos	54	72.00	182	82.40
	Total	P = 0.054			
E-cad	neg	46	34.60	130	33.30
	pos	87	65.40	260	66.70
	Total	P = 0.792			
N-cad	neg	78	60.50	255	64.40
	pos	51	39.50	141	35.60
	Total	P = 0.421			
Vim	neg	49	72.10	161	73.50
	pos	19	27.90	58	26.50
	Total	P = 0.813			
ALDH1	neg	51	98.10	156	97.50
	pos	1	1.90	4	2.50
	Total	P = 1.000 (F)			
Bcl-2	neg	111	86.00	354	91.00
	pos	18	14.00	35	9.00
	Total	p=0.107			
Apoptosis marker	neg	105	80.80	327	84.10
	pos	25	19.20	62	15.90
	Total	P = 0.384			
Ki-67	neg	93	66.90	313	74.70
	pos	46	33.10	106	25.30
	Total	P = 0.074			
Loxl-2	neg	17	12.00	36	8.70
	weakly pos	77	54.20	272	65.40
	pos	48	33.80	108	26.00
	Total	P = 0.059			
EGFR	neg	98	96.10	281	92.10
	pos	4	3.90	24	7.90
	Total	P = 0.257 (F)			

Categorization: CK5/6, CK14, vimentin, Bcl-2: neg – no expression, pos – weak to strong expression. CK8/18, CK19, E-cadherin, N-cadherin, EpCAM: neg – no or weak expression, pos – moderate or strong expression. ALDH1: neg – ALDH1 index score (i.e. intensity * % of the stained tumor cells) < mean, pos – ALDH1 index score >= mean. EGFR: neg – no to moderate expression, pos – strong expression. Loxl-2: : neg – no expression, weakly positive – weak expression, pos – moderate or strong expression. Ki67: neg - % of Ki-67-positive tumor cells < 1, pos – % of Ki-67-positive tumor cells >= 1. Apoptosis marker: neg – % of apoptosis marker-positive tumor cells < mean, pos – % of apoptosis marker-positive tumor cells >= mean.

Table S4. Molecular markers distribution. Distribution of the selected molecular signatures in tumor samples from unselected PCa patients cohort. Neg indicates negative expression, pos – positive expression.

**Molecular marker**		**N**	**%**
**CK5/6**	neg	601	88.00
	pos	82	12.00
	total	683	100
CK14	neg	477	70.50
	pos	200	29.50
	total	677	100
CK8/18	neg	260	35.90
	pos	465	64.10
	total	725	100
CK19	neg	366	53.80
	pos	314	46.20
	total	680	100
EpCAM	neg	100	25.60
	pos	290	74.40
	total	390	100
E-cad	neg	249	35.90
	pos	444	64.10
	total	693	100
N-cad	neg	442	64.00
	pos	249	36.00
	total	691	100
Vim	neg	259	71.20
	pos	105	28.80
	total	364	100
ALDH1	neg	261	97.00
	pos	8	3.00
	total	269	100
Bcl-2	neg	619	90.20
	pos	67	9.80
	total	686	100
Apoptosis marker	neg	580	84.70
	pos	105	15.30
	total	685	100
Ki-67	neg	533	71.40
	pos	213	28.60
	total	746	100
Loxl-2	neg	86	10.80
	weakly pos	486	60.80
	pos	180	22.50

	total	752	94.10
EGFR	neg	488	93.70
	pos	33	6.30
	total	521	100

Categorization: CK5/6, CK14, vimentin, Bcl-2: neg – no expression, pos – weak to strong expression. CK8/18, CK19, E-cadherin, N-cadherin, EpCAM: neg – no or weak expression, pos – moderate or strong expression. ALDH1: neg – ALDH1 index score (i.e. intensity * % of the stained tumor cells) < mean, pos – ALDH1 index score >= mean. EGFR: neg – no to moderate expression, pos – strong expression. Loxl-2: : neg – no expression, weakly positive – weak expression, pos – moderate or strong expression. Ki67: neg - % of Ki-67-positive tumor cells < 1, pos – % of Ki-67-positive tumor cells >= 1. Apoptosis marker: neg – % of apoptosis marker-positive tumor cells < mean, pos – % of apoptosis marker-positive tumor cells >= mean.

Table S5: Details of immunohistochemistry for proteins analysed in tumor cells.

Tumor marker	Antibody	Antigen retrieval	Dilution, incubation time	Detection system	Evaluation
CK5/6	clone D5/16B4, Dako, Denmark	0.01M citrate buffer (pH 6.0)	1:40, 25min / RT	DAKO LSAB 2 System-AP (Dako, Cytomation, Denmark)	intensity (no, weak, moderate, strong)
CK14	clone LL002, Dianova, Germany	0.01M citrate buffer (pH 6.0)	1:50, 25min / RT	DAKO LSAB 2 System-AP (Dako, Cytomation, Denmark)	intensity (no, weak, moderate, strong)
CK8/18	clone K8.8/DC10, Dianova, Germany	0.01M citrate buffer (pH 6.0)	1:40, 25min / RT	DAKO LSAB 2 System-AP (Dako, Cytomation, Denmark)	intensity (no, weak, moderate, strong)
CK19	clone KS19.1, Quartett, Germany	0.01M citrate buffer (pH 6.0)	1:500, 25min / RT	DAKO LSAB 2 System-AP (Dako, Cytomation, Denmark)	intensity (no, weak, moderate, strong)
EpCAM	NCL-ESA, Novocastra	1mg/ml trypsin 10 min / 37°C	1:75, 45min / RT	EnVision Kit, Rabbit/Mouse (Dako	intensity (no, weak, moderate, strong), % of the stained cells
E-cad	clone NCH-38,Dako, Denmark	Tris-EDTA (pH 9.0) in steamer	1:500, 25min / RT	LSAB/AP-Kit (Dako Cytomation, Denmark) / automated system (DAKO Autostainer, Denmark)	intensity (no, weak, moderate, strong)
N-cad	clone 6G11, Dako, Denmark	0.01M citrate buffer (pH 6.0) in steamer	1:100, 25min / RT	LSAB/AP-Kit (Dako Cytomation, Denmark) / automated system (DAKO Autostainer, Denmark)	intensity (no, weak, moderate, strong)
Vim	clone RV202, BD	0.01M citrate buffer (pH	1:100, 16h / 4°C	DAKO ChemMate Detection Kit Peroxidase/DAB,	intensity (no, weak, moderate,

	Pharmingen, USA	6.0) in steamer		Rabbit/Mouse (Dako, Denmark)	strong), % of the stained cells
ALDH1	44/ALDH1, BD Biosciences, US	0.01M citrate buffer (pH 6.0) in steamer	1:500, 16h / 4°C	DAKO ChemMate Detection Kit Peroxidase/DAB, Rabbit/Mouse (Dako, Denmark)	intensity (no, weak, moderate, strong), % of the stained cells
Bcl-2	clone124, DAKO	0.01M citrate buffer (pH 6.0) in steamer	1:250	automated system (DAKO Autostainer, Denmark) / alkaline phosphatase detection kit (Universal LSAB™ Kit/HRP, Rabbit/Mouse/Goat, Dako)	intensity (no, weak, moderate, strong)
Apoptosis marker	ApopTags (Chemikon, Germany) according to the manufacturer's protocol				% of the stained cells
Ki-67	clone MIB1, Dako Diagnostika, Germany	Target Retrieval Solution, Citrate pH 6.1 (Dako), in steamer	1:100, 25min / RT	LSAB/AP-Kit (Dako Cytomation, Denmark) / automated system (DAKO Autostainer, Denmark)	% of the stained cells
Loxl-2	courtesy of Prof. Katalin Csiszar	citrate buffer pH 6.0 for 20 min	1:600, 16h / 4°C	LSAB/AP-Kit (Dako Cytomation, Denmark) / automated system (DAKO Autostainer, Denmark)	intensity (no, weak, moderate, strong)
EGFR	E30, Dako	Proteinase K Ready-to-Use (Dako)	1:20, 16h / 4°C	EnVision Kit, Rabbit/Mouse (Dako)	intensity (no, weak, moderate, strong), % of the stained cells

Examples of evaluation roles and categorization of the results for patients:

Patient nr	VV Tumor fragment 1	VV Tumor fragment 2	LV Tumor fragment 1	LV Tumor fragment 2	minVV	maxVV	minLV	maxLV
1	no data	20	4	no data	20	20	4	4
2	6	no data	2	no data	6	6	2	2
3	19	no data	no data	2	19	19	2	2
4	10	19	1	2	10	19	1	2
5	23	27	0	0	23	27	0	0
6	15	11	0	0	11	15	0	0
7	46	10	3	0	10	46	0	3
8	16	8	2	4	8	16	2	4
9	8	7	1	1	7	8	1	1
10	24	15	0	0	15	24	0	0
11	20	8	0	3	8	20	0	3
12	12	10	3	0	10	12	0	3
13	6	12	2	no data	6	12	2	2
14	11	8	0	0	8	11	0	0
15	9	7	0	0	7	9	0	0
16	29	24	1	0	24	29	0	1
17	7	9	2	0	7	9	0	2
18	25	12	3	1	12	25	1	3
19	13	10	1	0	10	13	0	1
20	12	14	0	0	12	14	0	0

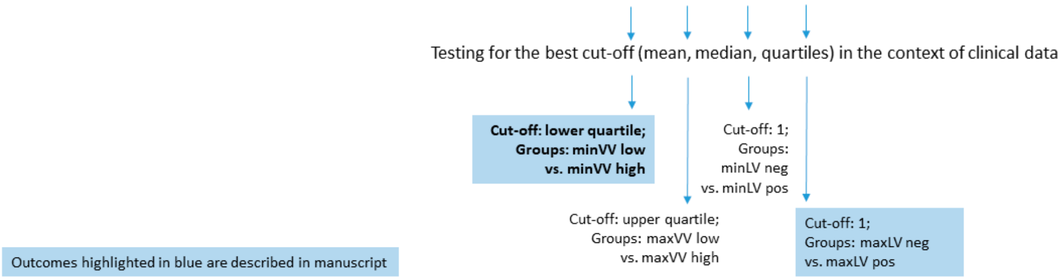

Figure S1: Scheme showing the process of evaluation of tumor samples and categorization of the outcomes. Scheme with some examples.

B) LV

Clinical and pathologic parameters	Status	Whole cohort, minLV				Hormone-naïve patients, minLV				Androgen deprivation therapy, minLV			
		neg		pos		neg		pos		neg		pos	
		n	%	n	%	n	%	n	%	n	%	n	%
Age (years)	< median (64)	114	51.60	87	52.10	93	52.00	72	50.70	21	50.00	15	60.00
	> = median (64)	107	48.40	80	47.90	86	48.00	70	49.30	21	50.00	10	40.00
	p value	*P* = 0.920				*P* = 0.824				P=0.427			
T status	T2	3	1.40	0	0.00	2	1.10	0	0.00	1	2.40	0	0.00
	T3a	102	46.20	76	45.80	84	46.90	70	49.30	18	42.90	6	25.00
	T3b	105	47.50	79	47.60	85	47.50	65	45.80	20	47.60	14	58.30
	T4	11	5.00	11	6.60	8	4.50	7	4.90	3	7.10	4	16.70
	p value	*p* = 0.439				*p* = 0.623				*p*=0.313			
N status	N0	201	94.40	155	95.10	162	94.20	130	94.20	39	95.10	25	100.00
	N1	12	5.60	8	4.90	10	5.80	8	5.80	2	4.90	0	0.00
	p value	*p* = 0.820 (F)				*p* = 1.000 (F)				*p*=0.522 (F)			
Gleason score	< 7	55	24.90	44	26.30	47	26.30	39	27.50	8	19.00	5	20.00
	7	139	62.90	107	64.10	112	62.60	90	63.40	27	64.30	17	68.00
	> 7	27	12.20	16	9.60	20	11.20	13	9.20	7	16.70	3	12.00
	p value	*p* = 0.706				*P* = 0.833				*p*=0.874			
Preoperative PSA	<4 ng/ml	27	12.60	15	9.20	26	14.70	12	8.60	1	2.70	3	12.50
	4-10 ng/ml	86	40.20	78	47.90	70	39.50	64	46.00	16	43.20	14	58.30
	10-20 ng/ml	60	28.00	53	32.50	50	28.20	48	34.50	10	27.00	5	20.80
	>20 ng/ml	41	19.20	17	10.40	31	17.50	15	10.80	10	27.00	2	8.30
	p value	*p*=0.060				*p*=0.087				*p*=0.132			
d'Amico scale	low risk	8	3.70	6	3.70	7	4.00	5	3.60	1	2.50	1	4.20
	intermediate low risk	51	23.80	38	23.50	44	25.30	33	23.90	7	17.50	5	20.80
	intermediate high risk	136	63.60	102	63.00	110	63.20	88	63.80	26	65.00	14	58.30
	high risk	19	8.90	16	9.90	13	7.50	12	8.70	6	15.00	4	16.70
	p value	*p*=0.991				*p*=0.971				*p*=0.949			
Preoperative ADT	no ADT	179	81.00	142	85.00	-	-	-	-	-	-	-	-
	ADT	42	19.00	25	15.00	-	-	-	-	-	-	-	-

	p value	*p*=0.298				-				-			
Biochemical recurrence	yes	63	28.60	39	23.50	46	25.80	35	24.80	17	40.50	4	16.00
	no	157	71.40	127	76.50	132	74.20	106	75.20	25	59.50	21	84.00
	p value	*p*=0.257				*p*=0.835				*p*=0.056 (F) *			
Tumor Focality	mono	38	17.30	25	15.10	32	18.00	20	14.20	6	14.30	5	20.00
	multi	182	82.70	141	84.90	146	82.00	121	85.80	36	85.70	20	80.00
	p value	*p*=0.560				*p*=0.362				*p*=0.734 (F)			

* Of note, the borderline correlation of maxLV^{neg} to BR in ADT-treated patients is probably a bias as minLV^{neg} (i.e. variable reflecting better the lack of LV) did not show any correlation to BR (data not shown). Therefore this outcome is not described within the manuscript.
